# Adversity Quotient Promotes Teachers’ Professional Competence More Strongly Than Emotional Intelligence: Evidence from Indonesia

**DOI:** 10.3390/jintelligence10030044

**Published:** 2022-07-21

**Authors:** Widodo Widodo, Irvandi Gustari, Chandrawaty Chandrawaty

**Affiliations:** 1 Social Science Education Department, Postgraduate Faculty, Universitas Indraprasta PGRI, Jakarta 12530, Indonesia; 2 Postgraduate School, Doctoral Program in Economics, Universitas Pancasiula, Jakarta 12640, Indonesia; irvandigustari@yahoo.com; 3 Faculty of Teacher Training and Education, Universitas Muhammadiyah Prof. Dr. HAMKA, Jakarta 12130, Indonesia; chandrawaty@uhamka.ac.id

**Keywords:** emotional intelligence, adversity quotient, organizational citizenship behavior, professional competence, teachers

## Abstract

Teachers’ professional competence has become a popular issue since the COVID-19 pandemic. Therefore, this study investigates teachers’ professional competence, in terms of emotional intelligence (EI), adversity quotient (AQ), and organizational citizenship behavior (OCB), when teachers need to deal with abnormal situations, such as the COVID-19 pandemic. Moreover, this study also seeks to explore the relationship of EI and AQ with teachers’ professional competence mediated by OCB. The research data were collected through a questionnaire using a Likert scale from 589 participants: elementary school teachers in Indonesia were chosen through accidental sampling. The data analysis used structural equation modeling (SEM), complemented by common method bias, correlational, and descriptive analysis. The result shows that EI, AQ, and OCB have a significant relationship with teachers’ professional competence. However, AQ more strongly promotes teachers’ OCB and professional competence than EI does. In addition, OCB mediates the relationship between EI and AQ with teachers’ professional competence. Accordingly, a new model regarding the relationship of EI and AQ with teachers’ professional competence mediated by OCB was confirmed. Therefore, it is suggested that teachers’ professional competence can increase through EI, AQ, and OCB. Hence, the new empirical model deserves to be discussed, adapted, and even adopted by practitioners and researchers to develop the professional competence of teachers in the future.

## 1. Introduction

Professional competence is essential for organizations, including that of teachers in a school organization context. Professional competence is proven to enhance teachers’ performance (Amalia and Saraswati 2018; Jie et al. 2020), work productivity (Nisa 2020), work effectiveness (Huda et al. 2020), and student achievement (Andriani et al. 2018). This indicates that professional competence is vital for a school organization, with implications for school graduates’ qualities and education qualities. That is, the professional competence of teachers can be a necessary condition for a nation’s human capital. Indonesia’s Human Capital Index (HCI) as of March 2020 (not counting the impact of the COVID-19 pandemic) is .54. This places Indonesia at 87th out of 174 countries. Indonesia’s Human Development Index (HDI) in the same year was even worse, ranking 111 out of 189 countries. The HCI and HDI might have been triggered by the condition of the professional competence of Indonesian teachers in previous years. This condition worsened during the COVID-19 pandemic, when teachers suddenly and without preparation had to carry out the online learning process due to the health protocol policy of physical distancing. At this time, the professional competence of teachers was tested. Therefore, it is important to investigate the issue of teachers’ professional competence during the COVID-19 pandemic, especially from the perspective of EI, which is the ability to understand, assess, feel, and express emotions appropriately and to relate the condition of oneself to others; AQ, which is the ability to face, respond to, and resolve life’s difficulties; and OCB, which involves discretionary behavior that a person performs voluntarily outside his formal role for the betterment of the organization. The three potentials (EI, AQ, and OCB) are very much needed of teachers, especially when dealing with abnormal situations such as the COVID-19 pandemic. This study focuses on investigating the relationship of EI, AQ, and OCB with teachers’ professional competence. It also seeks to explore the mediating role of OCB in the relationship of EI and AQ with teachers’ professional competence.

Conceptually, competence refers to an interrelated cluster of knowledge, skills, and abilities needed by an individual, team, or organization for effective performance (Hellriegel and Slocum 2011). Professionalism in the teaching context refers to meeting certain skill-related standards in education (Goodwin 2021). It reflects a set of functions, duties, and responsibilities within the education field based on skills acquired through specific education and training provided within the work field (Saguni et al. 2021). Grady et al. (2008) stated that a professional not only actively trains his wisdom in making important decisions related to his expertise but also tries to seriously develop his professional capacity. A professional is said to be competent when he acts responsibly and works effectively according to performance standards. That is, a professional must have adequate competence (Mulder 2014). Glickman et al. (2017) identified the stages of professional development through three learning steps, namely, orientation, integration, and refinement. In line with the above opinion, Mulder (2014) argues that professional competence is a generic, integrated, and internalized ability to realize sustainable performance effectively (including problem-solving, innovation, and creating transformation) in certain professional domains. In the educational context, primarily teaching, professional competence is the mastery of diverse and extensive teaching materials (Epstein and Hundert 2002). Hence, teachers’ professional competence refers to general characteristics that determine readiness and ability to adequately, independently, and responsibly carry out professional activities in a constantly changing social and professional environment, in order to be able to perform professional activities permanently and support personality-based self-development by understanding the social conditions of pedagogical activities (Orazbayeva 2016). Makovec (2018) mentions three indicators of teachers’ professionalism. First, teachers are proficient at the subject matter they teach, reviewing and updating knowledge of the subject. The second indicator relates to how teachers transmit their knowledge to students. It is related to the use of different didactic methods and takes account of class dynamics and a student’s age, prior knowledge, attitudes towards the subject, and characteristics. The third indicator is pedagogic. It is closely linked to the instruction, the interest in students’ personal issues and dilemmas, solving educational and disciplinary problems in or outside the class, and the teachers’ respectful, moral, decisive, and consistent actions, inside the class and among colleagues.

### 1.1. EI and Teachers’ Professional Competence

Teachers’ professional competence can be affected by EI. Previous studies by Papanikitas (2017) and Rahayu et al. (2018) show that self-awareness as an EI indicator influences professional competence. Other studies have also revealed that self-awareness and self-regulation are indicators of EI related to professionals in nursing practice (Kooker et al. 2007; Raghubir 2018), and professional boredom is included (Papathanasiou et al. 2021). This shows that EI indicators are an essential determinant for teachers’ professional competence. Jan and Anwar (2019) state that EI is a modern concept and a fundamental area of psychology and has a great influence on human life, including the student’s educational life. EI is also related to teaching effectiveness (Shahid et al. 2015) and academic performance (Sánchez-Álvarez et al. 2020). Silva and Coelho (2019) also demonstrated that EI affects creativity, increases task performance (Miao et al. 2018), and makes the project even more successful (Doan et al. 2020). Moreover, EI influences leadership range and effectiveness (Issah 2018; Lone and Lone 2018). Previous studies show that EI is crucial for individuals and organizational life, especially in a school organization. In addition, EI is related to psychological distress (Cheng et al. 2020), academic stress, and student achievement (García-Martínez et al. 2021).

EI relates to an individual’s ability, aptitude, recognition assignment, accurate appraisal, and management of their senses against other individuals and gatherings (Bradberry et al. 2009). It includes perceiving, valuing, and expressing emotions accurately, accessing and generating feelings that facilitate thinking, understanding emotions, having emotional awareness, regulating emotions, and promoting emotional and intellectual growth (Mayer and Salovey 1997). EI reflects the ability to feel, understand, and implement the sensitivity of power, and emotions act as a source of energy, information, and connections and influence that humanity (Cooper and Sawaf 1997). Furthermore, EI is an ability to become skilled at understanding particular emotional reactions, which can decide an individual’s ability to learn hands-on job-related social and emotional competencies (Zeidner et al. 2009). Individuals with a higher EI are more expected to regulate, understand, and control emotions well, in themselves and other individuals (Wijekoon et al. 2017). People with high EI are also better at perceiving their own and others’ behavioral causes, such as understanding why people are behaving in a certain manner and how to regulate their own and others’ behavior such that it leads to the growth and success of an individual as well as of those around them (Mahanta and Goswami 2020). In addition, EI is an ability to understand particular emotional reactions, which can determine an individual’s ability to learn hands-on, job-related social and emotional competencies (Zeidner et al. 2009).

A self-reported EI assesses the perceived efficacy of emotional processing (García-Martínez et al. 2021), including self-awareness, self-regulation, motivation, empathy, and relationship management (Goleman 2000). Self-awareness is knowing what one feels and using it as a reference to guide decision making, having a realistic assessment of one’s abilities, and having a reasonable degree of self-confidence. Self-regulation is handling emotions in a facilitating way, having awareness and delaying gratification in pursuing goals, and recovering well from emotional stress. Motivation is having preferences to drive and guide oneself toward desired goals, to take initiative and strive, and to improve and persevere in the face of setbacks and frustrations. Empathy is feeling what others are feeling and taking their perspective, as well as fostering connection and harmony with different people. Relationship management involves dealing with emotions in relationships properly and accurately by reading situations and social networks, interacting fluently, employing persuasion and leadership skills, and negotiating and resolving disputes to work together. These five indicators, self-awareness, self-regulation, motivation, empathy, and relationship management, if in high amounts, can enhance teachers’ professional competence in terms of the subject, didactic, and pedagogic indicators (Makovec 2018). For example, teachers who have robust self-regulation that is actualized by proactively facilitating students in the learning process can facilitate the process of transmitting and internalizing knowledge to students. Likewise, teachers who have high empathy and develop a deep concern for student problems, such as difficulty following lessons, can more easily help students solve personal problems. Therefore, the following hypothesis (H) can be formulated:

**Hypothesis** **1** **(H_1_).** *EI has a relationship with teachers’ professional competence.*

### 1.2. AQ and Teachers’ Professional Competence

Teachers’ professional competence can also be influenced by AQ. Although research regarding the effect of AQ on professional competence is still limited, Marashi and Fotoohi (2017) have indicated that AQ is related to professional development. Furthermore, an investigation conducted by Marashi and Rashidian (2018) demonstrated that AQ affects pedagogic success—an indicator of teachers’ professional competence. These studies, in addition to showing a limited relationship between AQ and professional competence, signal the need for new research that can more strongly determine the relationship between AQ and professional competence, especially from the perspective of teachers’ professional competence during the COVID-19 pandemic, to which AQ was a contributor. AQ also helps individuals strengthen their abilities and perseverance in facing the challenges of everyday life and achieving life satisfaction (Ablaña et al. 2016; Zhao et al. 2021), and this has an impact on performance (Tansiongco and Ibarra 2020). In the educational context, AQ is a determinant of learning behavior, learning outcome, graduate quality, and life satisfaction (Singh and Parveen 2018; Sigit et al. 2019; Puspitacandri et al. 2020), meaning that AQ is important for individual and organizational life. AQ refers to a persons’ ability or capacity to survive in the face of difficulties and to make efforts to resolve difficulties (Tigchelaar and Bekhet 2015; Hastuti et al. 2018). AQ has also been described as the capacity to deal with their adversities (Parvathy and Praseeda 2014), as an ability to face and overcome adversities and difficulties (Woo and Song 2015; Suryadi and Santoso 2017), and as the persistence of a person when dealing with obstacles to obtain success (Suryaningrum et al. 2020). Accordingly, AQ includes conquering obstacles, responding to challenges, and seizing opportunities.

According to Stoltz (2007), AQ can be measured by indicators: control, original ownership, reach, and endurance (CO2RE). Control is the ability to influence and control a situation. Original ownership reflects the ability to put feelings right, take risks, and fix problems. Reach is related to the ability to reach and limit problems so that one can participate in other areas of life. Endurance reflects the ability to face difficulties by creating new ideas so that the courage to solve various problems is built. All of these indicators, if in high degrees, can increase teachers’ professional competence in terms of the subject, didactic, and pedagogic indicators (Makovec 2018). As an illustration, teachers with a high degree of control, who can influence students and control a classroom atmosphere, will tend to be able to transfer knowledge to students more easily. In addition, teachers with a high degree of resilience, who can face adversity firmly while creating new creative ideas, will tend to be persistent in mastering the subject matter in many ways to carry out teaching tasks well. Therefore, the following hypothesis (H) can be formulated:

**Hypothesis** **2** **(H_2_).** *AQ has a relationship with teachers’ professional competence.*

### 1.3. OCB and Teachers’ Professional Competence

Teachers’ professional competence is also potentially influenced by OCB. Several previous studies have indicated that conscientiousness as an indicator of OCB is related to professionalism and competence (Finn et al. 2009; McLachlan et al. 2009; Jaya and Rukmini 2016; Trautwein et al. 2009). There is limited research on how OCB affects professional competence, and more research on the effect of OCB on professional competence is necessary, reviewing teachers’ professional competence during the COVID-19 pandemic, which required teachers’ OCB. Previous studies have revealed that OCB affects job performance (Yaakobi and Weisberg 2020; Yang and Chae 2021; Purwanto et al. 2022), teachers’ innovative behavior (Widodo and Gustari 2020), productivity (Barsulai et al. 2019), and organizational agility (Aval et al. 2017). This indicates that OCB is crucial for the survival and sustainability of individuals and organizations. Thus, it is important to investigate the link with teachers’ professional competence.

OCB is behavior outside the call of duty, such as collaboration to help others according to the social and psychological needs of the organization, which can help an organization’s survival (McShane and von Glinow 2020). Therefore, it is discretionary behavior outside of one’s formal role in the organization, for example, helping other employees who have not completed their tasks or simply showing support for the organization (Cascio 2016). OCB also refers to behaviors that support or improve cooperation in organizations and are not systematically or formally recorded to provide certain rewards. OCB reflects the contribution of all of an organization’s members, which can strengthen social relations between members and lead to extra-role behavior that is beneficial to the organization (Organ 2018).

Organ et al. (2006) mention five indicators to measure OCB: altruism, conscientiousness, sportsmanship, courtesy, and civic virtue. Altruism is related to the act of helping others, especially people who are experiencing personal difficulties, or related to others’ organizational tasks that have not been successfully completed. Conscientiousness refers to awareness and actions that move beyond organizational standards or expectations. Sportsmanship is related to tolerance for conditions in the organization that are less than ideal. Courtesy is an effort to maintain good relations with others to avoid conflicts or interpersonal problems. Civic virtue refers to a determination to take responsibility for the survival and success of the organization. If teachers demonstrate these indicators at a high level, such qualities can increase their professional competence in terms of the subject, didactic, and pedagogic indicators (Makovec 2018). For instance, teachers with high altruism will tend to facilitate the implementation of their pedagogical competencies. Therefore, the following hypothesis (H) can be formulated:

**Hypothesis** **3** **(H_3_).** *OCB has a relationship with teachers’ professional competence.*

### 1.4. The Relationship of EI and AQ with OCB

In addition to affecting professional competence, OCB is also influenced by EI. Scholars have concluded that EI significantly affects OCB (Miao et al. 2020). Similar studies have indicated that self-awareness as a part of EI relates to OCB (Rizki et al. 2019) and that the regulation of emotion as an indicator of EI affects OCB (Gan and Yusof 2018; Perveen et al. 2021). Furthermore, EI influences civic virtue, altruism, and conscientiousness as OCB indicators (Majeed et al. 2017; Meniado 2020; Mariyanti et al. 2021). This indicates that EI is an essential predictor of OCB. In practice, a teacher with high EI manifested in self-awareness, self-regulation, motivation, empathy, and relationship management (Goleman 2000) tends to have adequate OCB, reflected in altruism, conscientiousness, sportsmanship, courtesy, and civic virtue (Organ et al. 2006). For example, teachers with high empathy tend to have high levels of altruism.

OCB is also related to AQ. Siphai (2015) and Sobandi et al. (2021) have indicated that AQ affects OCB and that AQ is a vital antecedence for OCB. Teachers with high AQ, manifested in control, origin ownership, reach, and endurance (Stoltz 2007), tend to have adequate OCB, which is shown by altruism, conscientiousness, sportsmanship, courtesy, and civic virtue (Organ et al. 2006). As an illustration, teachers with high endurance tend to try hard to exceed organizational expectations (conscientiousness) and be responsible for their actions in organizational life (civic virtue). Thus, the following hypotheses can be formulated:

**Hypothesis** **4** **(H_4_).** *EI has a relationship with a teachers’ OCB.*

**Hypothesis** **5** **(H_5_).** *AQ has a relationship with a teachers’ OCB.*

### 1.5. The Mediation of OCB

Previous research on the mediating role of OCB on the relationship between EI and AQ with Teachers’ Professional Competence is still difficult to find. However, as has been shown in several previous studies, EI and AQ have an effect on OCB (i.e., Miao et al. 2020; Sobandi et al. 2021) and professional competence (i.e., Papanikitas 2017; Rahayu et al. 2018), while OCB has an effect on professional competence (i.e., McLachlan et al. 2009; Jaya and Rukmini 2016). This empirical fact clearly shows the mediating position of OCB between EI and AQ with professional competence. Thus, the following hypotheses can be formulated:

**Hypothesis** **6** **(H_6_).** *EI has a relationship with a teachers’ professional competence mediated by OCB.*

**Hypothesis** **7** **(H_7_).** *AQ has a relationship with a teachers’ professional competence mediated by OCB.*

## 2. The Current Study

The current study focuses on the relationship of EI, AQ, and OCB with teachers’ professional competence, specifically on the impact of the more dominant relationship between EI and EQ on OCB and professional competence and on new models regarding the mediating role of OCB in the relationship of EI and AQ with professional competence. SEM analysis and the voluntary support of teachers in Indonesia were used to achieve this goal. We endeavor to confirm the results of previous studies that are used as the basis for building the hypotheses of this study: the relationship of EI with OCB (Miao et al. 2020; Rizki et al. 2019; Gan and Yusof 2018; Perveen et al. 2021) and professional competence (Papanikitas 2017; Rahayu et al. 2018; Kooker et al. 2007; Raghubir 2018), of AQ with OCB (Siphai 2015; Sobandi et al. 2021) and professional competence (Marashi and Fotoohi 2017; Marashi and Rashidian 2018), and of OCB with professional competence (Finn et al. 2009; McLachlan et al. 2009; Jaya and Rukmini 2016; Trautwein et al. 2009). Finally, we hope to obtain empirical facts regarding the relationship of EI and AQ with professional competence, mediated by OCB, to build and build new models based on our research results.

## 3. Materials and Methods

### 3.1. Participants

The study participants (sample) were 589 elementary school teachers spread across four provinces in Indonesia, namely, Jakarta, Banten, West Java, and Central Java. They voluntarily filled out a questionnaire. Their profiles are presented in Table 1. The majority of participants were female (77.59%), aged 46–55 years (33.62%), had a bachelor’s degree (90.49%), were married (94.06%), and had 16 years of teaching experience (47.88%).

### 3.2. Procedure and Materials

This study used a quantitative approach with a survey method conducted using a Likert scale questionnaire with five alternative choices, namely, strongly disagree/never (score = 1), disagree/rarely (score = 2), neutral/sometimes (score = 3), agree/often (score = 4), and strongly agree/always (score = 5). This research occurred during the COVID-19 pandemic, which required participants and researchers to comply with health protocols, including physical distancing. Accordingly, the survey was conducted online using Google Forms shared via the WhatsApp application (in the teacher group’s WhatsApp network). The questionnaire was developed and compiled by the researcher himself based on the dimensions or theoretical indicators from experts. EI consists of self-awareness (SA), self-regulation (SR), motivation (Mot), empathy (Emp), and relationship management (RM) (Goleman 2000). AQ includes control (Con), origin ownership (OO), reach (Rea), and endurance (End) (Stoltz 2007). OCB includes altruism (Alt), conscientiousness (Con), sportsmanship (Spo), courtesy (Cou), and civic virtue (CV) (Organ et al. 2006). Furthermore, professional competence consists of subject (Sub), didactic (Did), pedagogic (Ped) indicators (Makovec 2018). Before using the survey, the questionnaire was first tested on 30 samples to determine its validity and reliability. Item validity was determined based on the corrected item-total correlation coefficient, while reliability was determined based on the alpha coefficient. For the EI questionnaire consisting of 10 items, the results show that the corrected item-total correlation coefficient is between .379 and .898, and the alpha coefficient is .917. The AQ questionnaire covered eight items, with a corrected item-total correlation coefficient between .418 and .671 and an alpha coefficient of .824. OCB covers 10 items, with a corrected item-total correlation coefficient between .520 and .820 and an alpha coefficient of .909. Professional competence consists of nine items, with a corrected item-total correlation coefficient between .538 and .782 and an alpha coefficient of .894. All items have a corrected item-total correlation coefficient >.361, and all variables have an alpha coefficient >.70, so it is valid and reliable as a research instrument (Van Griethuijsen et al. 2015; Hair et al. 2018).

In addition, to anticipate the anxiety of some researchers about the possibility of common method bias (CMB) problems in studies using one source, as in this study, procedural and statistical efforts were carried out. CMB is the magnitude of the discrepancy between the observed relationship and the true correlation between constructs (variables) generated by common method variance (CMV). CMV can increase the apparent correlation compared to the actual correlation. Therefore, CMV is a threat to producing valid and reliable research findings (Spector et al. 2019). Fuller et al. (2016) suggest the use of procedural and statistical improvements to control and minimize CMV. In this study, to detect CMV, the statistical mechanisms of Harman’s single-factor test (Malhotra et al. 2017) and the correlation matrix procedure (Tehseen et al. 2017) were used. Harman’s single-factor test shows that the total variance extracted by one factor is 40.112%, less than the recommended threshold of 50% (Kock 2020), and the correlation coefficient among constructs (variables) is less than .9 (Tehseen et al. 2017). This means there is no CMV (CMB) in the data of this study, so concerns about the occurrence of CMV (CMB) can be ignored.

### 3.3. Data Analysis

Data analysis was carried out with structural equation modeling (SEM), equipped with CMB, correlational, and descriptive analysis. To determine the significance of the direct correlation path coefficient, the Student’s *t*-test was used, while the Sobel (Z) test was used for the indirect correlation path coefficient (Abu-Bader and Jones 2021). CMB, descriptive, and correlational analysis was performed with SPSS version 22, while SEM analysis was performed with LisRel 8.80.

## 4. Results

### 4.1. Descriptive and Correlation Analysis

The results of the descriptive analysis of the four research variables (constructs) are as follows. The average values of the EI indicators from lowest to highest: SR = 7.86, RM = 7.87, Emp = 8.14, Mot = 8.52, and SA = 8.65. The average values of the AQ indicators from lowest to highest: OO = 8.23, End = 8.39, Rea = 8.71, and Con = 9.06. The average values of the OCB indicators from lowest to highest: Alt = 7.90, Cou = 8.09, CV = 8.14, Con = 8.35, and Spo = 8.71. The average values of the professional competence indicators from lowest to highest: Sub = 12.01, Did = 12.35, and Ped = 12.56. The standard deviation (std. dev) values of the EI indicators from lowest to highest: SA = .974, Mot = 1.079, Emp = 1.182, RM = 1.336, and SR = 1.453. The standard deviation (std. dev) values of the AQ indicators from lowest to highest: Con = 1.016, Rea = 1.080, End = 1.266, and OO = 1.313. The standard deviation (std. dev) values of the OCB indicators from lowest to highest: Con = 1.170, Spo = 1.262, CV = 1.286, Cou = 1.345, and Alt = 1.451. The standard deviation (std. dev) values of the professional competence indicators from lowest to highest: Did = 1.754, Ped = 1.775, and Sub = 1.919. As shown in Table 2, the standard deviation value is generally smaller than the mean value, so it reflects the data well. The correlation analysis on all indicators of the research variables also showed a significant relationship between the indicators at *p* < .01. This indicates that all indicators have reciprocal relationships.

### 4.2. Confirmatory Factor Analysis

The estimation of the measurement model carried out by confirmatory factor analysis is presented in Table 3. The factor loading values of all indicators and items that are ≥.3 are valid (Costello and Osborne 2005). That is, all indicators and items—as manifest variables—can measure all research variables as latent variables. Meanwhile, the reliability is determined based on the value of construct reliability (CR), variance extract (VE), and Alpha (α). The CR value of all variables is greater than .70, and the VE value of all variables is greater than .50, indicating good reliability and acceptable convergence (Hair et al. 2018).

### 4.3. Goodness of Fit

As shown in Table 4, the results of the goodness of fit (GOF) index show that 8 of the 11 measurements are categorized as good, while the other 3 (chi-squared values, sig., and RMSEA) are categorized as poor. According to Hair et al. (2018), the chi-square test is very sensitive to large sample sizes (>200), and this study involved 589 teachers. Therefore, the chi-square test, sig. probability, and the RMSEA values are considered ineffective. However, the GOF test results are still considered valid, because the other eight criteria tested met the requirements.

### 4.4. Hypothesis Testing 

**Figure 1:**
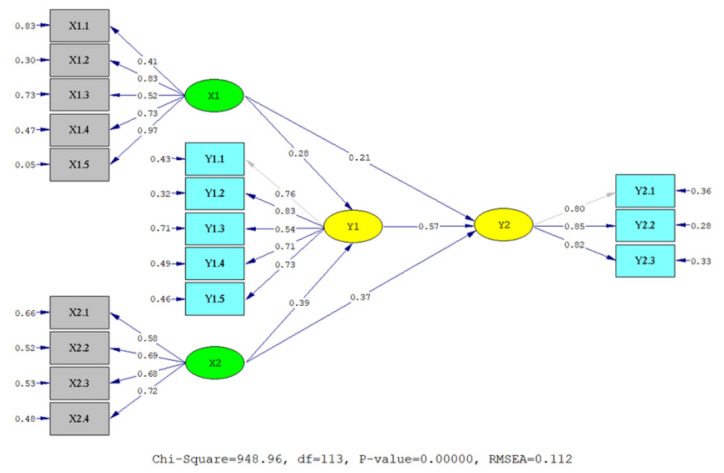
Standardized structural model. Note: X_1_ = EI, X_2_ = AQ, Y_1_ = OCB, Y_2_ = Teachers’ professional competence.

**Figure 2:**
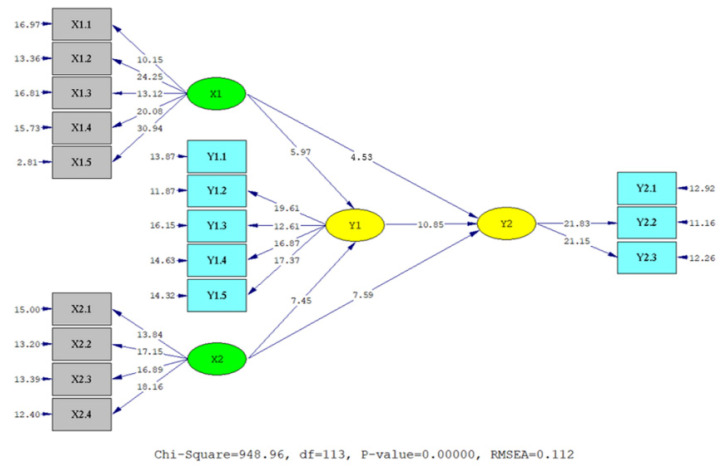
T-value structural model. Note: X_1_ = EI, X_2_ = AQ, Y_1_ = OCB, Y_2_ = Teachers’ professional competence.

The hypothesis testing results are summarized in Table 5 and visualized in Figure 1 and Figure 2. Empirical data supported all hypotheses (t value > t table at α =.01) by empirical data. In detail, EI, AQ, and OCB are significantly related to teachers’ professional competence (γ = .21, .37, and .57, *p* < .01). Likewise, EI and AQ also have a significant relationship with teachers’ OCB (γ = .28 and .39, *p* < .01). However, AQ has a stronger relationship with teachers’ OCB and professional competence than EI. The relationship of EI and AQ with teachers’ professional competence mediated by OCB is also significant. EI has a significant relationship with professional competence mediated by OCB (β = .16, *p* < .01). Likewise, AQ has a significant relationship with teachers’ professional competence mediated by OCB (β = .22, *p* < .01). However, AQ has a stronger relationship with teachers’ professional competence mediated by OCB than EI.

## 5. Discussion

This research shows that EI, AQ, and OCB have a significant relationship with teachers’ professional competence. This finding confirms that EI, AQ, and OCB are crucial determinants of teachers’ professional competence. The empirical result shows that teachers with high EI tend to have adequate professional competence. In other words, EI can improve teachers’ professional competence. This empirical result aligns with and confirms previous studies that suggest that EI affects teachers’ professional competence (Papanikitas 2017; Rahayu et al. 2018; Kooker et al. 2007; Raghubir 2018). EI is needed by teachers to ensure their professional competence. For example, if teachers have high levels of motivation, manifested in the desire to achieve certain goals, they take initiative and strive, improve and persevere in the face of setbacks, and are better able to master didactic competence. Likewise, teachers with high empathy can build pedagogic competencies. Overall, EI, with all its indicators, such as self-awareness, self-regulation, motivation, empathy, and relationship management, can enhance teachers’ professional competencies, in terms of subject, didactic, and pedagogic indicators.

This study also shows that AQ is significantly related to teachers’ professional competence. The empirical result shows that teachers with high AQ tend to have adequate professional competence; in other words, AQ can be relied upon to build professional competence. This is consistent with previous studies showing that AQ had a significant effect on professional competence (Marashi and Fotoohi 2017; Marashi and Rashidian 2018). In practice, AQ can help teachers build their professional competence. For example, teachers with high endurance can encourage their pedagogic competence. In addition, higher reach among teachers, manifested in reaching out and limiting problems so that one can participate in other areas of life, can drive them to mastery of the subject they teach. All AQ indicators, i.e., control, origin ownership, reach, and endurance, can increase teachers’ professional competencies, especially with respect to subject, didactic, and pedagogic indicators. 

This study also demonstrated that OCB has a significant relationship with teachers’ professional competence. This finding indicates that teachers with high OCB tend to solidify their professional competence; in other words, OCB can enhance teachers’ professional competence. This evidence is consistent with previous studies showing that OCB significantly influences professional competence (Finn et al. 2009; McLachlan et al. 2009; Trautwein et al. 2009; Jaya and Rukmini 2016). As an illustration, teachers with high levels of altruism and high levels of courtesy can easily achieve pedagogic competence. High conscientiousness among teachers, manifested in an effort to exceed the organization’s expectations, also drives them to quickly master subject content. This shows that all OCB indicators, i.e., altruism, conscientiousness, sportsmanship, courtesy, and civic virtue, can improve teachers’ professional competence, primarily with respect to subject, didactic, and pedagogic indicators.

This study also revealed that EI has a significant relationship with teachers’ OCB. The evidence shows that teachers with high EI tend to have strong OCB. In other words, EI can improve teachers’ OCB. This finding agrees with prior studies showing that EI is related to OCB (Gan and Yusof 2018; Rizki et al. 2019; Miao et al. 2020; Perveen et al. 2021). However, EI, which manifests in self-awareness, self-regulation, motivation, empathy, and relationship management (Goleman 2000), is a predisposition that allows teachers to demonstrate high levels of altruism, conscientiousness, sportsmanship, courtesy, and civic virtue (Organ et al. 2006). For instance, high empathy among teachers can help them to have adequate levels of altruism. Likewise, teachers with high levels of relationship management can easily and quickly have high levels of sportsmanship and courtesy. In sum, higher self-awareness, self-regulation, motivation, empathy, and relationship management can improve teachers’ altruism, conscientiousness, sportsmanship, courtesy, and civic virtue.

This study also indicated that AQ has a significant relationship with OCB. This confirms that teachers with adequate AQ tend to have strong OCB, which means that AQ can be a vital asset for teachers in terms of developing their OCB. These findings confirm studies in which AQ was found to be related to OCB (Siphai 2015; Sobandi et al. 2021). AQ indicators, i.e., control, origin ownership, reach, and endurance (Stoltz 2007), can enhance OCB, manifested in altruism, conscientiousness, sportsmanship, courtesy, and civic virtue (Organ et al. 2006). In practice, teachers with high endurance are more likely to make an effort to exceed the school’s expectations. In addition, teachers’ control capacity also promotes their sportsmanship.

In sum, AQ has a stronger relationship with teachers’ OCB and professional competence than EI. Hence, AQ needs to be given more attention and priority in the context of its causal relationship with OCB and professional competence.

This study presents findings that led to a new model regarding the relationship between EI and AQ with teachers’ professional competence mediated by OCB. This model can be adapted and adopted for the development of teachers’ professional competence based on EI, AQ, and OCB across various locations, sectors, organizations, and contexts.

This study indicates the strength of EI, AQ, and OCB in affecting teachers’ professional competence. Therefore, teachers’ EI, AQ, and OCB should be continuously improved using an appropriate strategy or approach. For instance, the teachers should independently and consciously enhance their EI, AQ, and OCB by reading the relevant literature or discussing, role-playing, and applying the newest concepts, methods, approaches, or techniques of EI, AQ, and OCB. School principals should encourage teachers to participate in training programs specifically designed to improve teachers’ EI, AQ, and OCB. They need to initiate and facilitate training programs of EI, AQ, and OCB involving expert instructors. The provided training material, followed by the methods and training media used, should lead to the mastery of knowledge and skills regarding EI, AQ, and OCB. However, AQ should be highlighted because of its dominant role in affecting OCB and task performance.

As a complement, this study also simulates the relationship of AQ and OCB with professional competence in the reverse order: professional competence as a predictor of AQ and OCB. The results show that professional competence has a significant relationship with AQ (β = .65, *p* < .01) and OCB (β = .74, *p* < .01). This indicates that AQ, OCB, and professional competence have mutual relationships. This fact needs to be responded to carefully by researchers. Moreover, other simulation results indicate that when AQ is positioned as a mediator, the relationship between AQ and OCB becomes insignificant (β = .07, *p* < .05). Accordingly, future research must be done to carefully position each of these variables according to the context.

## 6. Conclusions

Teachers’ professional competence, including teachers’ performance, work productivity, and work effectiveness, is essential for school organizations. Moreover, it can have implications for school graduates’ qualities, education qualities, and a nation’s human capital. This study found that EI, AQ, and OCB have a significant relationship with teachers’ professional competence. Furthermore, AQ has a stronger relationship with teachers’ OCB and professional competence than EI. In addition, OCB mediates the relationship of EI and AQ with teachers’ professional competence. Accordingly, a new model regarding the relationship of EI and AQ with teachers’ professional competence mediated by OCB was confirmed. This model provides a theoretical contribution to the OCB mediation model of the relationship of EI and AQ with professional competence as well as complement and strengthen theoretical buildings still lacking, for example, the relationship between EI, AQ, and OCB with professional competence. Hence, researchers can respond to, adopt, or develop this model in future research. Meanwhile, in practice, this finding contributes to teachers’ performance and implicates student achievement and school performance. Therefore, it is suggested that teachers’ professional competence can be improved through EI, AQ, and OCB. AQ should be a priority because of its dominant role in affecting OCB and task performance. For example, practitioners, primarily teachers, should independently and consciously improve their EI, AQ, and OCB by reading relevant literature or discussing, role-playing, and applying the latest concepts, methods, approaches, or techniques with respect to EI, AQ, and OCB. Meanwhile, school principals should encourage teachers to participate in training programs specifically designed to improve teachers’ EI, AQ, and OCB.

## 7. Limitations and Future Research

Although it has been carried out carefully and according to scientific procedures, this research is not without its shortcomings and has several limitations. First, it does not adopt all of the theoretical indicators/dimensions available in the literature. Therefore, future research should adopt indicators/dimensions not included in this study. Second, this study does not explore the empirical facts behind the relationship of EI and AQ with teacher professional competence directly or indirectly mediated by OCB. Therefore, further research should seek to respond to this limitation by relying on mixed methods—quantitative and qualitative analysis. Finally, this study only uses a single data source (teachers). Hence, future research can replicate the findings of this study, particularly with respect to OCB and professional competencies, by adding other data sources (participants), such as students, colleagues (teachers), and school principals.

## Figures and Tables

**Table 1:** The research participants’ profile.

Profile	Amount	Percentage
Gender		
Male	132	22.41
Female	457	77.59
Age		
≤25 years	16	2.72
26–35 years	121	20.54
36–45 years	173	29.37
46–55 years	198	33.62
≥56 years	81	13.75
Education		
Diploma (D3)	44	7.47
Bachelor (S1)	533	90.49
Postgraduate (S2)	11	1.87
Doctoral (S3)	1	0.17
Status		
Married	554	94.06
Unmarried	35	5.94
Experience		
≤5 years	73	12.39
6–10 years	66	11.21
11–15 years	168	28.52
≥16 years	282	47.88

**Table 2:** Descriptive statistics and correlation matrices.

Variables	Mean	Std. Dev	1	2	3	4	5	6	7	8	9	10	11	12	13	14	15	16	17
EI																			
1. SA	8.65	.974	1.00																
2. SR	7.86	1.453	.34 **	1.00															
3. Mot	8.52	1.079	.50 **	.35 **	1.00														
4. Emp	8.14	1.182	.48 **	.46 **	.60 **	1.00													
5. RM	7.87	1.336	.37 **	.83 **	.48 **	.71 **	1.00												
AQ																			
6. Con	9.06	1.016	.28 **	.19 **	.27 **	.24 **	.14 **	1.00											
7. OO	8.23	1.313	.23 **	.29 **	.24 **	.33 **	.31 **	.42 **	1.00										
8. Rea	8.71	1.080	.29 **	.24 **	.29 **	.28 **	.27 **	.42 **	.49 **	1.00									
9. End	8.39	1.266	.31 **	.30 **	.34 **	.39 **	.31 **	.37 **	.50 **	.49 **	1.00								
OCB																			
10. Alt	7.90	1.451	.37 **	.32 **	.32 **	.48 **	.43 **	.21 **	.29 **	.18 **	.36 **	1.00							
11. Con	8.35	1.170	.35 **	.26 **	.38 **	.44 **	.33 **	.34 **	.25 **	.22 **	.38 **	.65 **	1.00						
12. Spo	8.71	1.262	.23 **	.13 **	.25 **	.21 **	.15 **	.27 **	.17 **	.21 **	.29 **	.29 **	.49 **	1.00					
13. Cou	8.09	1.345	.24 **	.22 **	.36 **	.38 **	.28 **	.31 **	.20 **	.15 **	.31 **	.55 **	.55 **	.43 **	1.00				
14. CV	8.14	1.286	.23 **	.22 **	.36 **	.36 **	.27 **	.26 **	.24 **	.17 **	.27 **	.53 **	.58 **	.39 **	.62 **	1.00			
Professional Competence																			
15. Sub	12.01	1.919	.35 **	.20 **	.41 **	.41 **	.27 **	.33 **	.35 **	.35 **	.40 **	.44 **	.49 **	.35 **	.33 **	.44 **	1.00		
16. Did	12.35	1.754	.35 **	.19 **	.39 **	.37 **	.26 **	.38 **	.32 **	.33 **	.43 **	.42 **	.54 **	.40 **	.36 **	.43 **	.70 **	1.00	
17. Ped	12.56	1.775	.37 **	.23 **	.36 **	.40 **	.29 **	.35 **	.28 **	.34 **	.42 **	.51 **	.53 **	.39 **	.44 **	.47 **	.64 **	.69 **	1.00

** *p* < .01.

**Table 3:** The measurement model results.

Variables	Indicators	Items	Factor Loading		CR	VE	α
			Item	Indicator			
EI	SA	I really understand my capabilities as a teacher.	.81	.91	.947	.643	.917
		I believe I can solve various problems that arise at school.	.85				
	SR	I use the power of emotions to fight for life goals that have not been achieved.	.79	.87			
		I know the right way to express my feelings.	.75				
	Mot	I actively take the initiative to help students solve problems.	.67	.84			
		I am enthusiastic about facing various challenges.	.72				
	Emp	I can feel what other people feel.	.84	.94			
		I easily build social relationships with different people.	.88				
	RM	I consider social situations when interacting with other people.	.82	.96			
		I prioritize a persuasive approach in resolving disputes.	.86				
AQ	Con	I put the situation in context.	.58	.80	.847	.512	.824
		I control every situation optimally.	.68				
	OO	I put my feelings fairly.	.69	.93			
		I am responsible for all the risks of my actions.	.61				
	Rea	I am sincere if I can only solve some of life’s problems.	.59	1.02			
		I take the time to explore the side of life that has been neglected.	.54				
	End	I am ready to face various difficulties in life.	.80	.82			
		I am determined to solve any complex life problems.	.61				
OCB	Alt	I sincerely share my knowledge with other teachers.	.77	.87	.885	.543	.909
		I am willing to help solve various problems at school.	.62				
	Con	I use my work time as efficiently as possible.	.48	1.28			
		I usually finish tasks faster than usual.	.58				
	Spo	I see the shortcomings in school as an opportunity to do good.	.64	.76			
		I try my best to help solve unfinished school problems.	.75				
	Cou	I am proactive in establishing good relations with other teachers who have different views.	.76	.81			
		I am willing to give in to avoid conflict.	.82				
	CV	I am actively involved in various additional activities at school.	.45	1.11			
		I prioritize school interests over personal matters.	.68				
Professional Competence	Sub	I master the subject matter that I teach.	.60	1.02	.833	.509	.894
		I evaluate the subject matter routinely.	.65				
		I update the subject matter regularly.	.59				
	Did	I use various teaching methods.	.51	1.14			
		I consider the characteristics of students in delivering the subject matter.	.61				
		I take into account class dynamics in teaching.	.56				
	Ped	I pay attention to students’ learning interest in teaching.	.53	.99			
		I take into account the actual condition of the student’s personality in the learning process.	.67				
		I focus on solving various learning problems faced by students.	.65				

**Table 4:** Goodness-of-fit statistics.

Goodness of Fit Index	Cut of Value	Results	Information
Absolute fit measure			
Chi-square	X^2^ < X^2^ table	948.96	Poor
Sig. Probability	*p* > .05	.00	Poor
GFI	≥.09	.84	Good
RMSEA	≤.08	.11	Poor
Incremental fit measures			
NFI	>.90	.93	Good
NNFI	>.90	.92	Good
AGFI	>.90	.98	Good
CFI	>.90	.94	Good
RFI	>.90	.91	Good
Parsimony fit measures			
Normed chi-square	1–2 or <3	1.75	Good
PNFI	0–1	.77	Good

**Table 5:** Hypothesis testing results.

Hypothesis	β/γ	T/Z Value	Decision
H_1_: EI (X_1_) and teachers’ professional competence (Y_2_)	.21 **	4.53	Supported
H_2_: AQ (X_2_) and teachers’ professional competence (Y_2_)	.37 **	7.59	Supported
H_3_: OCB (Y_1_) and teachers’ professional competence (Y_2_)	.57 **	10.85	Supported
H_4_: EI (X_1_) and OCB (Y_1_)	.28 **	5.97	Supported
H_5_: AQ (X_2_) and OCB (Y_1_)	.39 **	7.45	Supported
H_6_: EI (X_1_) and teachers’ professional competence (Y_2_) mediated by OCB (Y_1_)	.16 **	10.89	Supported
H_7_: AQ (X_2_) and teachers’ professional competence (Y_2_) mediated by OCB (Y_1_)	.22 **	9.82	Supported

** *p* < .01.

## Data Availability

Not applicable.
